# Predisposition to boredom and anxio-depressive disorders among nurses : A cross-sectionl descriptive study

**DOI:** 10.1192/j.eurpsy.2025.1975

**Published:** 2025-08-26

**Authors:** I. Kacem, A. Ghenim, O. Ajmi, M. Bouhoula, N. Belhadj, A. Chouchane, S. Ben Abderrahmen, A. Aloui, N. Mrizak

**Affiliations:** 1 Departement of Occupational Medicine; 2 Department of psychiatry , Farhat Hached Academic hospital, Sousse , Tunisia

## Abstract

**Introduction:**

Bore-out affects three times as many employees as burn-out. It results from a lack of activity during work, and the healthcare sector, due to its monotony, promotes the emergence of these disorders

**Objectives:**

Study the predisposition of hospital nurses to boredom, and investigate a possible relationship between this predisposition and their anxiety-depression profile.

**Methods:**

This is a cross-sectional descriptive study conducted in April 2022 involving all nurses at the Sahloul University Hospital in Sousse. We used a pre-established questionnaire that included two validated tools: the Boredom Proneness Scale (BP) and the Hospital Anxiety and Depression Scale (HAD).

**Results:**

A total of 65 nurses took part in this study. The mean age was 36.62 ± 4.82 years and the sex ratio was 0.38. According to the BP Scale, 13 nurses were inclined towards boredom (20%). A predisposition to boredom has been observed among staff with less than 8 years of seniority (p= 0.001), those with no extracurricular activities (p= 0.020), those with a moderate or severe workload (p= 0.008), and those whose profession had no impact on their relationships with others (p= 0.012).The depression score on the HAD scale was moderate (32%), average (20%), and severe (12%). The HAD scale revealed that 29% of people had mild anxiety, 23% had moderate anxiety, and 12% had severe anxiety. No statistically significant relationship was found between the boredom disposition scale and the HAD scale.

**Conclusions:**

Bore-out can worsen anxiety and depressive disorders by causing boredom and frustration. It is crucial to promote an engaging and varied work environment to protect employees’ mental health.

**Disclosure of Interest:**

None Declared

